# Emergent Attentional Bias Toward Visual Word Forms in the Environment: Evidence From Eye Movements

**DOI:** 10.3389/fpsyg.2018.01378

**Published:** 2018-08-03

**Authors:** Jing Zhao, Hang Yang, Xuchu Weng, Zhiguo Wang

**Affiliations:** ^1^Institute of Psychological Sciences, Hangzhou Normal University, Hangzhou, China; ^2^Zhejiang Key Laboratory for Research in Assessment of Cognitive Impairments, Hangzhou, China; ^3^Center for Cognition and Brain Disorders, Hangzhou Normal University, Hangzhou, China

**Keywords:** environmental print, reading development, visual attention, eye tracking, reading ability

## Abstract

Young children are frequently exposed to environmental prints (e.g., billboards and product labels) that contain visual word forms on a daily basis. As the visual word forms in environmental prints are frequently used to convey information critical to an individual’s survival and wellbeing (e.g., “STOP” in the stop sign), it is conceivable that an attentional bias toward words in the environment may emerge as the reading ability of young children develops. Empirical findings relevant to this issue, however, are inconclusive so far. The present study examines this issue in children in the early stages of formal reading training (grades 1, 3, and 5) with the eye-tracking technique. Children viewed images with word and non-word visual information (environmental prints) and images with the same words in standard typeface on a plain background (standard prints). For children in grade 1, the latency of their first fixations on words in environmental prints was longer than those in standard prints. This latency cost, however, was markedly reduced in grades 3 and 5, suggesting that in older children an attentional bias toward words has emerged to help filter out the non-word visual information in environmental prints. Importantly, this attentional bias was found to correlate moderately with word reading ability. These findings show that an attentional bias toward visual word forms emerges shortly after the start of formal schooling and it is closely linked to the development of reading skills.

## Introduction

**FIGURE 1:**
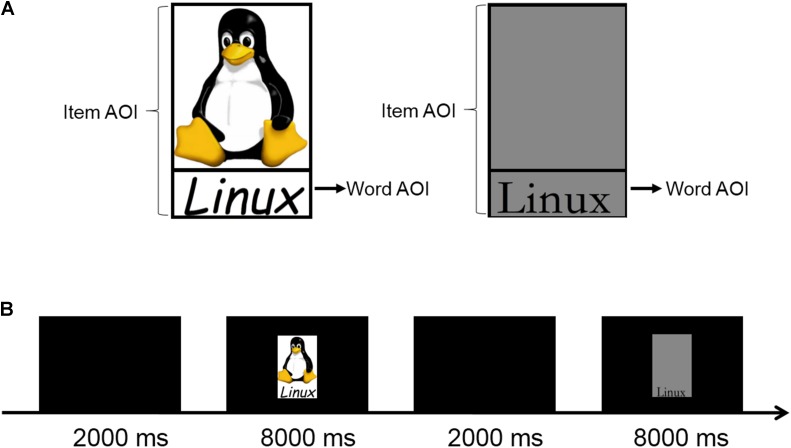
**(A)** Sample environmental and standard prints, and their item- and word-AOIs. **(B)** Sequence of events in a mini-block of trials in the free viewing task. For copyright issues, the logos cannot be used in this illustration. Therefore, we use the Linux logo instead to illustrate the definition of word and item AOIs in environmental and standard prints. The Linux log (the penguin in the above illustration) was created with GIMP by Larry Ewing (lewing@isc.tamu.edu). The Linux logo now used in the illustration is for illustration purpose only; it was not used in our study. The Linux logo, is copyrighted to Larry Ewing, and can be used for any purpose, provided that the copyright holder is properly attributed.

Young children are exposed to a vast amount of printed texts long before formal education begins (Neumann et al., 2012). These environmental prints (e.g., traffic signs and billboards) contain not only visual word forms but also salient non-word visual information, such as colors, logos, and artful typefaces (for an example, see **Figure 1**). Environmental prints are functional, ubiquitous, and salient, providing a valuable perceptual experience of visual word forms to young children (Horner, 2006; Vukelich et al., 2008). In a short-term training program, Cronin et al. (1999) found that children learned words that were part of environmental prints (e.g., McDonald’s) better than control words (e.g., Monster), even when all words were presented in a standard typeface. This is no surprise, as children are frequently exposed to words in environmental prints and they may have acquired knowledge of these words through statistical learning (e.g., Saffran et al., 1996; Kirkham et al., 2002). Furthermore, previous findings seem to suggest that frequent exposure to visual word forms also shapes the way we sample visual information. The most compelling demonstration of elevated attentional priority of visual word forms is the failure to ignore alphanumeric information (e.g., letters and numbers), even when they are “irrelevant” within the context of an experimental task, such as the Stroop Task (e.g., Flowers and Stoup, 1977). Recent studies have also shown that texts in real-world scenes attract more attention than regions of similar size and position in free viewing tasks (e.g., Cerf et al., 2009; Wang and Pomplun, 2012). Because visual word forms in the environment typically convey information that is critical to the survival and wellbeing of an individual, it is no surprise that an attentional bias toward them is developed early in life. When does this attentional bias emerge in the early years of development? Is it linked to the development of reading skills? The empirical findings relevant to these questions are inconclusive so far.

Previous work examining words in environmental prints have shown that young children may rely on non-word information (e.g., logos) to recognize the words in environmental prints (Share and Gur, 1999; Blair and Savage, 2006). In an early study, Masonheimer et al. (1984) found that, when presented in black and white in a standard typeface, most 3–5 years old children (94%) could not read words contained in environmental prints. When some letters in these words were substituted (e.g., replacing P’s in PEPSI with X’s), children usually failed to notice the substitutions (Masonheimer et al., 1984; see Neumann et al., 2012, for a discussion). A recent study by our group also showed that 3–4 years old children rely primarily on graphical cues to name words contained in environmental prints (Zhao et al., 2014). These results seem to suggest that young children pay more attention to non-word visual information (e.g., logos) than to visual word forms in environmental prints.

As a powerful tool in reading research, eye-tracking allows real-time monitoring of the direction of gaze and thus provides a direct measure of whether visual word forms in environmental prints are preferentially attended by young children. In a recent study, Neumann et al. (2014) asked preschool children (age range: 3–5 years) to view either environmental prints with both words and non-word visual information, or images with the same words but no non-word visual information. They found that children did direct gaze to words in environmental prints, however, these words were fixated less frequently and for shorter durations compared to the same words that were not embedded in non-word visual information. The latency of the first fixation with words was longer for environmental prints than that for images without non-word visual information. These observations suggest that, while salient non-word visual elements in environmental prints help to attract young children’s attention, they may draw attention away from the visual word forms contained in environmental prints. In a follow-up study, Neumann et al. (2015) confirmed that pre-readers, who scored low on a sight vocabulary test, paid more attention to words in word-salient versus picture-salient environmental prints. By contrast, for those who scored higher on the same sight vocabulary test, attention to words in environmental prints was relatively unaffected by the relative salience of words, indicating that children may develop an attentional preference for visual word forms as their reading ability develops.

It is important to note that the above-mentioned studies have exclusively examined children learning to read alphabetic scripts. It is unclear whether these findings can be generalized to different writing systems (i.e., logographic scripts, such as Chinese). The visual word form of a Chinese character corresponds to its phonology at the syllable level; there is no direct grapheme to phoneme mapping. Previous studies have shown that knowledge about visual word forms (e.g., visual features and orthographic regularities) is more important in reading Chinese than in reading alphabetic scripts (Tan et al., 2005). The results of a recent study found that Chinese children begin to utilize the orthographic information of words in environmental prints in ages 3–4 (Zhao et al., 2014), indicating that exposure to visual word forms in the environment may be more important to reading development in logographic writing systems. The primary purpose of the present study was to examine the impact of environmental prints in Chinese children. More specifically, we aim to address: (a) when does the attentional bias toward visual word forms emerge in the early stages of schooling, and (b) whether this attentional bias is linked to the reading skills of young children.

In addition to examining the attentional bias toward visual word forms in a population reading a logographic script, the present study also helps to extend our knowledge about this attentional bias in school-aged children. The subjects of most previous studies were pre-readers (Masonheimer et al., 1984; Cronin et al., 1999; Neumann et al., 2014; Zhao et al., 2014) and beginning readers (Neumann et al., 2015), who had received little formal training in reading. To examine whether the development of reading ability changes the way young children select visual information from the environment, it is necessary to systematically characterize the attentional bias toward words in environmental prints in school-aged children, whose reading skills greatly improve as the years of formal education increases.

As a first step of this endeavor, the present study examined the attentional bias toward visual word forms in environmental prints between ages 7–11 (grades 1–5), during which children quickly master reading (Shu and Anderson, 1997; Liu et al., 2010; Tong and McBride-Chang, 2010). The methodology of the present study was similar to that in previous work (e.g., Neumann et al., 2014, 2015). Children viewed environmental prints and control images while their gaze was monitored with a video-based eye-tracker. As noted above, the use of eye tracking is necessary to directly examine whether children preferably attend to visual word forms in environmental prints. To measure children’s reading ability, a word recognition test developed for school-aged children was also administrated (Shu et al., 2003).

## Materials and Methods

The research protocol reported here was approved by the human research reviewing committee of the Institute of Psychological Sciences, Hangzhou Normal University. Written informed consent was obtained from the parents or other legal guardians of all children who participated the present study.

### Participants

Sixty-five children were recruited from a local primary school. They were native Mandarin speakers, and all had normal or corrected-to-normal vision. They were grouped based on the number of years of formal education they had received: 21 were first-graders (mean age = 7.25 years, *SD* = 0.51; 10 boys), 18 were third-graders (mean age = 9.32 years, *SD* = 0.36; 10 boys), and the remaining 26 were fifth-graders (mean age = 11.36 years, *SD* = 0.42; 15 boys).

### Materials

We first selected twenty full-color product logos that children frequently encounter in their daily life (i.e., environmental prints, hereafter called EPs; see **Figure 1A**, for an example). The names of the manufacturers and other small printed words were first removed from these images to ensure only one salient word was present in each of the environmental prints. These images measured between 10.85°× 13.30° and 28.62°× 20.94° (visual angle), at a viewing distance of 65 cm. The relative positioning of the words and the non-word visual elements was not fixed in the environmental prints. The words appeared in the lower region of eight images, appeared in the upper region of seven images, and appeared in the right region of five images.

Similar to previous studies (Neumann et al., 2014, 2015), control images with the same words as the EPs were also tested. The control images were used to examine how fast the participants directed attention to these words when non-word visual information was also present. In these control images, words were presented in a standard Chinese typeface (Song), in black color, against a gray background. For convenience and to facilitate comparison to previous work, these images will be referred to as “standard prints” (SPs; see **Figure 1A**, for an example). Words in SPs had the same size, orientation, and position as those in the corresponding EPs.

### Apparatus

All stimuli were presented on a 19-inch CRT monitor, at a viewing distance of about 65 cm (maintained by using a chinrest). Stimulus presentation and response registration were controlled by a Mac Mini running scripts generated with Experiment Builder (SR Research, Ottawa, ON, Canada). An EyeLink 1000 eye-tracker (SR Research, Ottawa, ON, Canada) was used to monitor and record the participant’s gaze direction. The tracking accuracy of the eye-tracker was reported to be 0.25° or better and the sampling rate of the eye-tracker was set to 500 Hz.

### Word Recognition Test

A word recognition test developed for primary school children (Shu et al., 2003) was used to assess the participants’ reading ability. Specifically, the test items (150 Chinese characters) were arranged in increasing difficulty and children were asked to read out the test items one by one. The score is the number of correctly identified Chinese characters. The maximum score possible on this test is 150 (see Shu et al., 2003, for a detailed description).

### Free Viewing Task

The main task was a free viewing task, in which we assessed whether children had an attentional bias toward visual word forms (see **Figure 1B**). This task was similar to that used in Neumann et al. (2014). All test images were presented against a black background. A total of 20 EPs and 20 SPs were presented in five mini-blocks of eight trials, with resting periods in between. A standard 9-point calibration of the eye tracker was performed at the beginning of each mini-block.

Each mini-block had four EP items and their corresponding SP items. Items within each mini-block were presented in a random order, with the constraint that no more than two items of the same type (EP or SP) were presented in sequence, and that an EP and its corresponding SP were never presented in sequence. The five mini-blocks were counterbalanced across participants. Each item was presented for 8000 ms, followed by a 2000 ms interval during which a black screen was presented. The participants were naïve with respect to the purpose of the experiment, and they were instructed to simply view the screen.

## Results

### Processing of Eye Movement Data

Similar to Neumann et al. (2014), areas of interest (AOIs) were first defined for each EP and SP image (see **Figure 1A**). The AOIs were manually drawn around the word and picture areas of the EP and SP items. The word AOI was a single rectangular region closely enclosing all words, and the item AOI was the display region occupied by the entire image. Because EPs and their corresponding SPs were identical in size and shape, each pair of EP and SP stimuli had identical word and item AOIs. Fixations outside the item AOIs were not considered in the analysis.

The eye movement data was analyzed with Data Viewer (SR Research, Ottawa, ON, Canada). Fixations following blinks were removed because the eyes rotate when the eyelids move. Fixations close to each other were merged (amplitude threshold = 1.5°), and those with durations shorter than 50 ms were not considered in the analysis.

Three dependent measures were considered: (a) percentage of fixations in word AOIs, i.e., how often fixations landed in word AOIs; (b) percentage of fixation duration in word AOIs, i.e., the ratio between the total fixation time in word AOIs and that in item AOIs; and (c) latency of the first fixation in word AOIs, i.e., the time interval between item onset and the first fixation in the word AOI. The first two dependent measures were ratios, which properly controlled for the between-subject variations in total fixations and fixation durations (Neumann et al., 2014). In rare occasions where a word AOI was never fixated during the 8000 ms viewing time (3.40% of the trials), the percentage of fixations and fixation durations in word AOIs was set to 0%, and the latency of the first fixation in word AOI was set to 8000 ms.

The dependent measures were first extracted for each item (EP or SP). The participants’ means were then submitted to 2-way mixed ANOVAs, with variables age (7, 9, vs. 11 years) and Image Type (EP vs. SP).

### Attentional Bias Toward Visual Words

**FIGURE 2:**
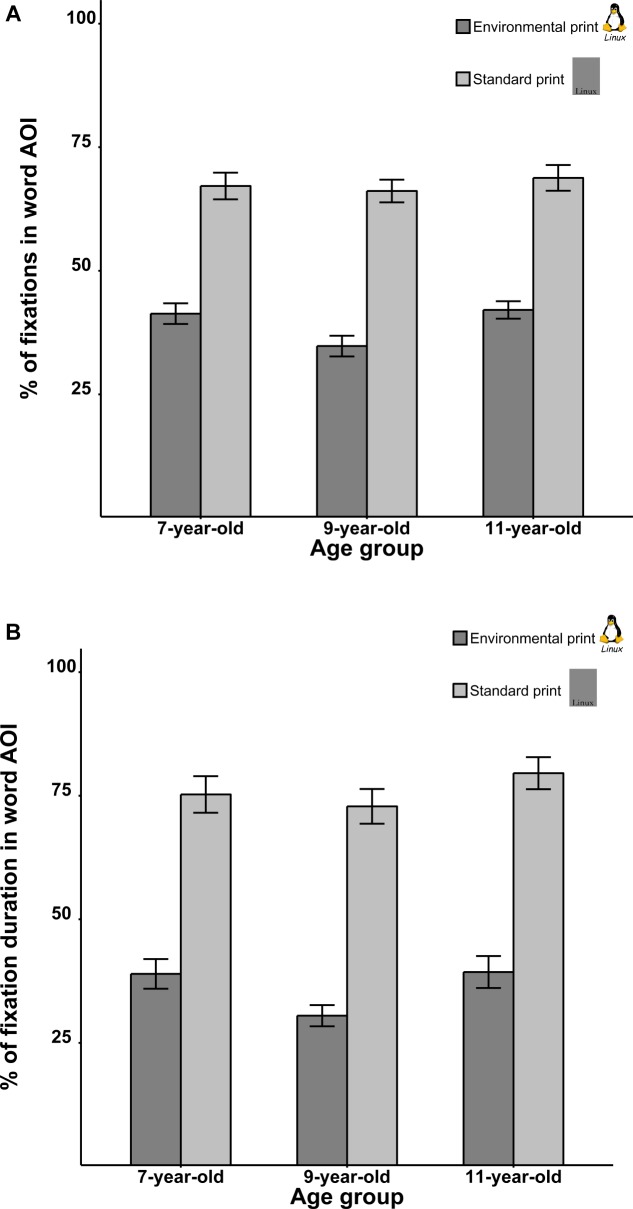
Percentage of fixations **(A)** and fixation durations **(B)** for the word AOI in environmental prints and standard prints. Error bars denote ± SEM.

The percentages of fixations in word AOIs in EPs and SPs are presented in **Figure 2A**. A mixed ANOVA on this dependent measure, with age (7, 9, and 11 years) as a between-subject variable and Image Type (EP vs. SP) as a within-subject variable, revealed a significant main effect of Image Type, *F*(1,62) = 368.62, *p* < 0.001, $\eta_{\text{p}}^{2}$ = 0.86; word AOIs in SPs were fixated more often than those in EPs. This observation was no surprise given that words were the only visual elements visible in SPs (see **Figure 1A**). There was no main effect of Age, *F*(2,62) = 1.80, *p* = 0.17, $\eta_{\text{p}}^{2}$ = 0.06 and there was no interaction between Image Type and Age, *F*(2,62) = 1.27, *p* = 0.29, $\eta_{\text{p}}^{2}$ = 0.04.

The percentage of fixation duration in word AOIs in EPs and SPs are presented in **Figure 2B**. A mixed ANOVA revealed a significant main effect of Image Type, *F*(1,62) = 347.50, *p* < 0.001, $\eta_{\text{p}}^{2}$ = 0.85, with word AOIs in SPs being fixated longer than those in EPs. There was no main effect of Age, *F*(2,62) = 2.23, *p* = 0.12, $\eta_{\text{p}}^{2}$ = 0.07 and there was no interaction between Image Type and Age, *F*(2,62) = 0.65, *p* = 0.52, $\eta_{\text{p}}^{2}$ = 0.02.

**FIGURE 3:**
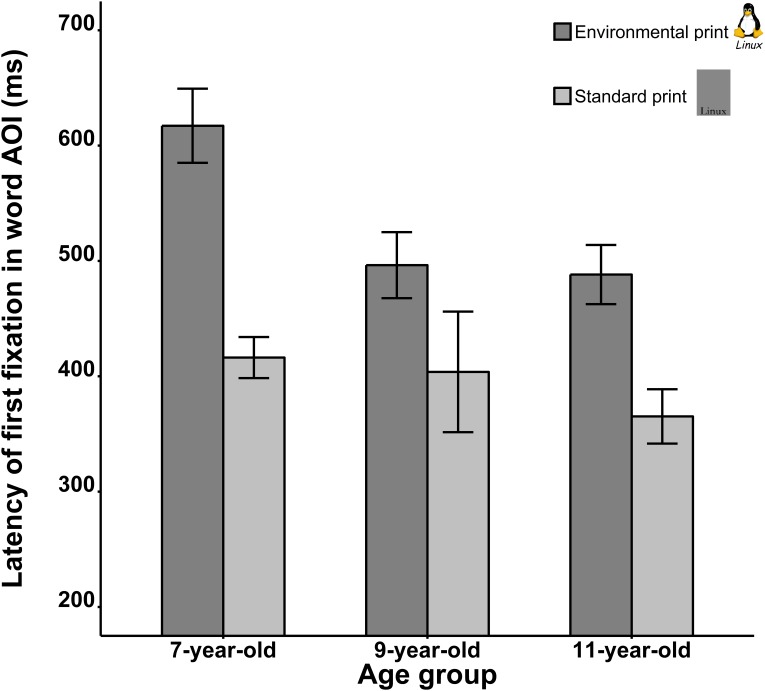
Mean latencies of the first fixation in word AOIs in EPs and SPs across three age groups. Error bars denote ± SEM.

Latency of the first fixation in word AOI. To determine whether visual words captured attention early, we examined the latency of the first fixation in word AOIs. Mean latencies of the first fixation in word AOIs are presented in **Figure 3**. Results of the mixed ANOVA revealed significant main effects for Image Type, *F*(1,62) = 69.60, *p* < 0.001, $\eta_{\text{p}}^{2}$ = 0.53, and Age, *F*(2,62) = 3.41, *p* = 0.04, $\eta_{\text{p}}^{2}$ = 0.10. As shown in **Figure 3**, these main effects were observed because the latency of the first fixation in word AOIs was longer for EPs than SPs, and generally decreased as age increased. Importantly, the two-way interaction was significant, *F*(2,62) = 3.58, *p* = 0.03, $\eta_{\text{p}}^{2}$ = 0.10. The latency difference between EPs and SPs was larger in 7 years old children (201 ms) than that in 9 years (93 ms), *t*(37) = 2.57, *p* = 0.01, and 11 years (123 ms) old children, *t*(45) = 1.99, *p* = 0.05, whereas no difference was found between 9 and 11 years old children, *t*(42) = 0.75, *p* = 0.46.

Words in EPs were accompanied by non-word visual information (e.g., logos), and thus the latency of the first fixation in word AOIs was expected to be longer for EPs compared to SPs. The fact that this latency difference was much smaller in the 9 and 11 years old children suggests an attentional bias toward words has emerged in older children, which effectively reduces the ability of salient non-word visual information (e.g., color, logo, and typeface) in absorbing attention.

### Reading Ability and the Attentional Bias Toward Words

The Chinese word recognition test scores of each group are presented in **Table 1**. As noted above, the first fixation in word AOIs was shorter in SPs, as compared to EPs. This latency difference reflects how fast gaze is directed to the words when no-word visual elements are present. A reduction of this latency difference is evidence that the words take less time to win the competition for selection, i.e., become more salient or attention-absorbing. As shown in **Figure 3**, the latency of the fixations in word AOIs for SPs was largely unchanged between ages seven and nine, whereas that for the EPs was markedly reduced. This observation clearly shows that the latency difference is unlikely the result of the development in information processing speed.

**Table 1:** Mean scores on the Chinese word recognition test.

Group	Reading score
7-year-old	29.24 (16.86)
9-year-old	110.67 (18.42)
11-year-old	129.62 (9.33)

*SD of the mean is given in parentheses.*

When the attentional bias toward visual word forms was measured with the above noted latency difference between SPs and EPs, it was found to robustly correlate with the word recognition test scores, *r* = −0.31, *p* = 0.018; the attentional bias toward visual word forms was stronger for children with higher word recognition scores. This negative correlation persisted when age was controlled for in the correlation analysis (*r* = −0.30, *p* = 0.02). These results clearly show that children with higher level of reading ability were faster at directing attention to the visual word forms in EPs.

## Discussion

The present study examined whether the attentional bias toward visual word forms emerges with the development of reading skills in the early stages of schooling. Consistent with previous work (Neumann et al., 2014), words in environmental prints were fixated less frequently and for shorter durations than words in control images that contained no non-word visual information. These results clearly show that the non-word visual information in EPs is highly attention-absorbing. However, the school-aged children studied in the present study appeared to allocate more attentional resources to words in EPs than pre-school children. Over 30% of the fixations were directed to words in school-aged children (the present study), whereas in pre-school children (Neumann et al., 2014), only about 20% of the fixations were allocated to words in EPs. The latency of the first fixation in word AOIs in EPs was much shorter in school-aged children (538 ms, present study) than that in pre-school children (about 3500 ms, Neumann et al., 2014).

The most important finding of the present study was that an attentional bias toward words emerges as children’s reading ability quickly develops between ages seven and nine. For children who just started formal schooling (7 years old), the latency of the first fixation on words surrounded by non-word visual information (i.e., in EPs) was longer than that of the same words presented in standard typeface, against a plain background (i.e., in SPs). This latency difference, however, was markedly reduced for 9 and 11 years old children, suggesting an attentional bias toward words has developed, perhaps to disengage their attention from non-word visual information (e.g., color, logo, and typeface). Without this attentional bias, the eyes are easily attracted by salient non-word visual information, as seen in the 7-year-old children tested in the present study. When children are 9–11 years old, an attentional bias toward words has developed, such that the eyes are directed to visual word forms in EPs much more efficiently. Further analysis showed that this attentional bias toward words, as manifested in the latency difference of the first fixation on words between EPs and SPs, correlated moderately with children’s reading ability. This observation suggests a close relationship between reading ability and the attentional bias toward visual word forms in the environment. Nevertheless, we would like to point out that this attention-based theory may not be the only sensible explanation of the present results. We did not include a post-test interview to assess the strategy or mental states of the children during the free viewing task. It is possible that higher level cognitive processes were also in play. This may explain why we failed to observe any statistically significant difference in measures like total dwell time and total fixation count. Nevertheless, we believe the latency of the first fixation in word AOIs did reflect fairly early and low-level attentional processes. As shown in **Figure 3**, the latency of the first fixation in word AOIs was generally below 600 ms. That is barely two gaze shifts, given that people normally make three to four rapid gaze shifts a second in tasks like visual search, scene viewing and reading (e.g., Rayner, 1998).

One may suggest that the critical correlation observed here may simply reflect the general development of cognitive abilities with age. This alternative explanation is unlikely to be true. As clearly shown in **Figure 3**, the latency of the first fixation in word AOIs in SPs decreased with age, possibly because attentional orienting becomes more efficient as the age of the children increases. However, a more drastic decrease was observed in the EPs, within which salient non-word visual elements are competing for selection with words. The use of SPs as control stimuli has to some extent controlled for this potential confound. Importantly, a robust correlation between the attentional bias and children’s reading ability was still observed when age was controlled for in the analysis. To experimentally rule out this possibility, future studies need to include a control condition where visual word forms in environmental prints are replaced with physically comparable non-word stimuli (e.g., stroke combinations, Zhao et al., 2012). If the observed attentional bias toward words was the result of development in general cognitive ability, one should fail to observe a correlation between reading ability and the fixation latency disadvantage for non-word control stimuli.

It is also worthwhile to note that, in contract to the present results, a previous study failed to observe a correlation between reading ability and the attentional bias toward words in preschool children learning to read alphabetic scripts (Neumann et al., 2014). One possible explanation of this discrepancy is that the present study examined a totally different writing system, i.e., a logographic script (Chinese). Chinese characters have several unique characteristics, notably, the visual word form of a Chinese character corresponds to its phonology at the syllable level and thus, no grapheme to phoneme conversion is needed. The exposure to environmental prints may be particularly helpful to Chinese children in acquiring visual orthographic knowledge, which is crucial for reading Chinese (Tan et al., 2005). In contrast, the exposure to environmental prints may be less helpful to children learning alphabetic scripts, in which phonological rather than orthographic decoding skills are more fundamental. Alternatively, it could be that the participants in Neumann et al.’s (2014) study had little word knowledge and their reading scores were largely at floor level. As a result, no correlation between reading ability and attentional bias toward words was observed.

Previous work has shown that overlearned visual word forms have higher attentional priority (e.g., Cerf et al., 2009; Wang and Pomplun, 2012), however, it is unclear whether this attentional bias emerges quickly as the reading ability of children develops (Masonheimer et al., 1984; Cronin et al., 1999; Neumann et al., 2012, 2014, 2015; Zhao et al., 2014). The present study addressed this issue by systematically characterizing the attentional bias toward visual word forms in environmental prints in school-aged children, whose reading ability greatly improves as the years of formal education increases. The present work extends previous studies on pre-readers (Masonheimer et al., 1984; Cronin et al., 1999; Neumann et al., 2014; Zhao et al., 2014) and beginning readers (Neumann et al., 2015) to school-aged children, who have received intensive formal training in reading. Our results suggest that the development of reading skills is accompanied by an elevated attentional priority of visual word forms, providing support to the idea that the known attentional bias toward visual word forms is developed through learning (see Cerf et al., 2009, for a discussion). However, a few limitations of the present study also should be noted. Firstly, the present study adopted a cross-sectional design to reveal a developmental trend of the attentional bias toward words in the environment; a longitudinal study is needed to confirm these findings. Secondly, reading-related cognitive abilities (e.g., visual-orthographic, phonological, and semantic skills) and other cognitive skills (e.g., intelligence) were not measured, so it remains unclear whether there are other factors that mediate the relation between the attentional bias toward words and reading ability. Thirdly, a control condition with physically comparable non-word stimuli may help to preclude the influence of the general development of cognitive abilities with age. This additional control condition was not examined in the present study.

To summarize, the present study shows an attentional bias toward visual word forms in the environment quickly emerges between ages 7 and 9, and that this attentional bias is correlated with children’s reading ability. Future longitudinal studies to clarify whether there is causality between reading development and this attentional bias are strongly encouraged.

## Author Contributions

All authors designed the experiment. HY collected the data. HY, JZ, and ZW analyzed the data. JZ drafted the manuscript. ZW and XW provided critical revisions. All authors approved the final version of the manuscript for submission.

## Conflict of Interest Statement

The authors declare that the research was conducted in the absence of any commercial or financial relationships that could be construed as a potential conflict of interest.
